# The course of metastatic prostate cancer under treatment

**DOI:** 10.1186/2193-1801-3-725

**Published:** 2014-12-10

**Authors:** Aslan Demir, Kursat Cecen, Mert Ali Karadag, Ramazan Kocaaslan, Levent Turkeri

**Affiliations:** Department of Urology, Kafkas University Faculty of Medicine, Kars, Turkey; Department of Urology, Marmara University Faculty of Medicine, Istanbul, Turkey

**Keywords:** Metastatic prostate cancer, Treatment modality, Course

## Abstract

The first-line management of metastatic prostate cancer is hormonal therapy. However, resistance to this treatment will emerge within an average of 24 months. Our purpose was to determine the course of metastatic prostate cancer under treatment.

A total of 56 patients who were diagnosed with metastatic prostate cancer were enrolled. As initial management, 3 kinds of hormonal therapy consisting of bilateral orchiectomy (BSO) alone, BSO + anti-androgene (AA) and LH-RH + AA were applied. The patients were followed until the emergence of hormone resistance. Serum PSA levels at the time of first diagnosis, post-treatment nadir PSA levels, time to nadir PSA, time to hormonal resistance and PSA levels at hormonal resistance were assessed, retrospectively. The localization and number of metastases and the survival term from the beginning of the emergence of hormone resistance until death were investigated

No significant differences could be established between the groups. The mean time to reach hormone refractory status was 30.3 months for the whole study group. The average term of survival was 42.7 months for the whole group. Distance metastases were found in 8 patients during follow-up.

There were no statistical differences between the groups in terms of treatment modalities applied for metastatic prostate cancer. Patients with androgen independent prostate cancer demonstrated progression despite chemical or surgical castration, and had poor prognosis. Initial hormonal therapy failed after an average of 2 years in metastatic prostate cancer.

**Electronic supplementary material:**

The online version of this article (doi:10.1186/2193-1801-3-725) contains supplementary material, which is available to authorized users.

## Background

Most of the prostate cancers (PCa) are, at least at the beginning, endocrine-dependent tumors. Therefore, hormonal therapy is still a first-choice treatment (Chen et al. 2008). The first effects to hormonal treatment with medical or surgical castration are quite considerable, with rapid biochemical responses, as evaluated by declines in levels of the serum marker, prostate-specific antigen (PSA) (Di Lorenzo et al. 2010; Chi et al. 2009). However, most patients showing a first response to hormonal therapy for PCa will progress to a castration-insensitive phase of the disease that continues a much poorer prognosis (Chi et al. 2009).

The true is that androgens have an important role in the whole clinical course of Pca, even when a patient meets castration-resistance criteria (Pinto 2013). Luteinizing hormone (LH) and follicle-stimulating hormone (FSH) are of considerable roles in PCa progression. Specifically, LH expression in PCa tissues has been associated with metastatic disease with a poor prognosis, while FSH has been related to stimulate prostate cell growth in hormone-refractory PCa (Kluth et al. 2013). Endocrine therapy is the standard treatment for metastatic PCa, but in its progess an androgen independence status can occurs. As endocrine management, primary androgen deprivation therapy (ADT) is the standard of care, usually with a long-releasing LHRH analogue or antagonist (Pagliarulo et al. 2012).

The aim of our study was to determine the course of the disease in patients with initial metastatic PCa.

## Results

A total of 56 patients were enrolled. In this study, the median age of the patients was 70 (min. 59 – max. 93). BSO alone was performed in 12 patients (21.7%), adjunctive antiandrogen therapy was given to 18 patients (32.05%) and LH-RH analogues combined with antiandrogen therapy were given to 26 patients (46.1%). No statistically significant differences could be established between the groups according to the parameters of our study (Table 1). The mean PSA (ng/ml) values before treatment were 171, 383.7, and 626.5, respectively. The Gleason Scores (GS) of all groups were 7. The mean time to the emergence of hormone refractory status was found to be 30.3 months for the whole study group. The average survival was 42.7 months (min. 9-max. 120) in the whole group. Mean survival time (month) were 45, 45.25, and 34.25, respectively. Distance metastases were found in 8 patients during follow-up. The prognosis of these patients was poor.

**Table 1:** **The summary of the results of each treatment group**

Parameters	BSO n = 12		BSO + AA n = 18		LH-RH + AA n = 26		WHOLE GROUP n = 56	
	Median	Mean	Median	Mean	Median	Mean	Median	Mean
The PSA (ng/ml) values before teratment	91	171	143	383,7	115	626,5	110	423,8
Gleason score	7	7,33	7	7,5	7	7,52	7	7,4
The nadir PSA (ng/ml) after treatment	1,3	31,01	3,6	37,47	2,4	7,87	2,71	21,68
The time to the nadir PSA (months)	3	5,9	4,5	6,45	8,5	9,27	6	7,44
The time to hormonal resistance (months)	18,7	32,77	17	26,48	21	27,3	20	30,28
The PSA (ng/ml) at the hormonal resistance	103,5	124,2	54	163,7	38,5	890,6	54	489,9
Survival (months)	25	45	47	45,25	32	34,35	34	42,67

All p values >0,05.

## Discussion

Monotherapy and combined therapy are the hormonal management modalities. The first one contains castration or anti-androgens treatment, and the second one includes maximum or complete androgene blockage (MAB or CAB) with castration (medical or surgical) and anti-androgens. However, a refractory status to these treatment will progress within an average of 2 years. Poor prognosis and death from the disease within 18–24 and 22 months have been reported by some researchers in the literature (Crawford et al. 1989; Kelly et al. 2010) but in the beginning, estimates of median survival for patients with hormone-refractory PCa were 7–12 months (Hussain et al. 1994). When the patients progress in front of continued androgen ablation, they are named to have hormone-refractory prostate cancer or metastatic castrate-resistant prostate cancer (mCRPC) (Martel et al. 2003).

Most patients with PCa at the organ confined level have an excellent prognosis after radical prostatectomy and/or radiation therapy. A significant fraction (20%-40%) of patients who undergo primary therapy experience biochemical progression, and 30–70% of those with biochemical relapse progress to the metastatic status within 10 years after local management (Pound et al. 1999). Usually, patients with hormone-refractory PCa may apply with symptoms associated with metastases, especially bone, lymph nodes and other urinary metastases, but the first sign is generally, an increased PSA levels despite to the treatment (Martel et al. 2003; Sammon et al. 2013).

The population of PCa with distant metastasis is heterogeneous (European Association of Urology Guidelines 2013). One of the poor prognostic factors was the PSA level greater than 65 ng/ml (Chi et al. 2009). According to the results of our study, mean PSA (ng/ml) values before treatment were 171, 383.7, and 626.5, respectively. The GS of all groups in our study were 7.

The choice between orchiectomy and oestrogens are the standard treatment for advanced PCa (Murphy et al. 1983; Sharifi 2009). It is well known that the initial development of PCa is dependent on androgens; therefore, the first-line management of this situation is endocrine management. First-line treatment for advanced PCa is ADT, usually with agents that inhibates gonadotropins through a pituitary mechanism. Gonadotropin-releasing hormone agonists and antagonists both inhibates gonadal release of testosterone, although their activity profiles differ (Murphy et al. 1983; Kluth et al. 2013).

ADT down-regulates the androgen receptor (AR) in the tumor but the response in advanced disease is not permanent, a progression may occur as CRPC. Although serum testosterone levels decrease significantly with ADT, CRPC development largely depends on AR activity (Sharifi 2009). Secondary hormonal therapies are then often used to further lessen AR-driven. These secondary hormonal managements either further deplete adrenal or intratumoral androgens synthesis, or directly and competitively antagonize AR (Sharifi 2009).

The median survival time for patients with metastatic androgen-independent PCa is approximately 1 or 2 years (Hussain et al. 1994; Kelly et al. 2010). In most studies in the literature, no statistically significant differences have been reported between the results of different hormonal treatment modalities that are performed in metastatic PCa. The results of our study are similar to those in the literature. The Median and mean survival time (month) in our study were 25/45, 47/42.25, and 32/34.35, respectively.

Robinson et al. compared the alternatives of orchiectomy and maximal androgen blockage with 1 mg diethylstilbestrol (DES) in 1995 and declared no difference in terms of progression rate and survival between the two groups (Robinson et al. 1995). In another study conducted by Mikkola et al., orchiectomy and parental polyestradiol phosphate were compared and no difference was reported in terms of progression and survival follow-up for 2 years (Mikkola et al. 1998). Laufer et al. found no benefits in the use of combined androgen blockade (CAB) with medical or surgical castration (Laufer et al. 2000). In addition, a non-steroidal antiandrogen for metastatic PCa was investigated in 20 randomized trials by Crawford et al. which led to the finding that there is a 5% improvement in the percentage of men surviving 5 years (30% vs. 25%) with combined androgen blockade with nonsteroidal antiandrogens, as well as improvements in progression-free survival at 1 year (Crawford et al. 1989).

The patients with metastatic PCa ought to be informed of the potential benefits, toxicities and out-of pocket expenditures about managements modalities (Schmitt et al. 2001; Prostate Cancer Trialists’ Colloborative Group 2000). Gastrointestinal, ophthalmological and hematological side effects are worse with CAB. Although LHRH analogues and non-steroidal anti-androgens have the highest estimated quality-adjusted survival, there is an increased cost of more than US$1 million per quality-adjusted life-year for CAB over orchiectomy alone (European Association of Urology Guidelines 2013). In a SWOG study published in 1998, although there was no statistical difference, using MAB decreased the risk of mortality about 9% compared with orchiectomy alone. In addition, two meta-analyses have declared that there is an increase in response rate using MAB with non-steroidal antiandrogen compared to mere castration (Prostate Cancer Trialists’ Colloborative Group 2000; Caubet et al. 1997). On the other hand, the same studies mentioned that there was also an increase in time to progression when using MAB with non-steroidal antiandrogen compared with other managements (Caubet et al. 1997; Glashon & Robinson 1981).

Fossa et al. have mentioned responses to flutamide in some patients in whom previous endocrine managements failed, and found that this correlated with increased survival (Fossa et al. 1990). On the other hand, Andrew et al. have established that orchiectomy as a secondary hormonal management following relapse on antiandrogen does produce a response in terms of PSA level and symptoms in some patients (Andrew et al. 2001). In the only published randomized controlled trials (RCT), there was no significant difference in OS for flutamide monotherapy compared in bone metastatic patients with a PSA <100 ng/ml. But at a higher PSA, flutamide was inferior. (European Association of Urology Guidelines 2013).

Bicalutamide, 150 mg once daily, it has been compared to medical or surgical castration in two large prospective RCTs with identical study designs, including patients with locally advanced or metastatic PCa; the analysis found that in metastatic patients, there was an improvement in OS with medical or surgical castration, although the difference in median survival between the groups was only 6 weeks (Tyrrell et al. 1998).

According to our results, there is no statistical difference in terms of survival between the hormonal treatment modalities that have been applied for metastatic PCa in the literature, but we must pay attention to the status of each patient with advanced PCa, such as psychological state and tolerance to the side effects of the treatment modalities, since the quality-of-life is paramount. Antiandrogen therapy has remained the mainstay in the treatment of metastatic PCa. To reduce the testosterone level in blood, both oestrogens and bilateral orchiectomy can be use effectively (Martel et al. 2003). However, oestrogens have obvious disadvantages and particularly cardiovascular, even life-threatening, side effects need to take into the consideration (Glashon & Robinson 1981). On the other hand, the underestimation of the psychological side effects of bilateral orchiectomy is possible. Therefore, the advantages of LH-RH analogues have led to the new views in the management of PCa. Superactive LH-RH analogues effectively reduce plasma testosterone to castrate levels (Jacobi & Wenderoth 1982). Therefore, these drugs can be considered as the first choice alternatives to oestrogens or orchiectomy, but we believe that not only is cost-effectiveness an important issue that should be considered for patients who do not have any health insurance, but the socio-cultural level of patients is also important in terms of accommodation to the treatment. Namely, if the socio-cultural and economic level of the patient is low, then BSO may be the first choice for treatment.

In addition to these findings, the development of a hormone-refractory state in patients with advanced PCa is inevitable. Initial androgen ablation can provide, on average, 18 months of biochemical response. There has been an interest in utilizing antiandrogen monotherapy in these patients initially to preserve quality-of-life.

If the biochemical relapse does occur under treatment of antiandrogen monotherapy, to know whether further hormonal manipulation will provide any benefit is important. Scher et al. have declared that patients who had a biochemical relapse on maximal androgen blockade and then had a response to antiandrogen withdrawal, had a further response to the addition of a different antiandrogen (Scher et al. 1997). It has been suggested that stopping of androgen deprivation before the progression of androgen resistant situation would mean that any subsequent tumor growth would be sustained by the proliferation of androgen-sensitive stem cells. The stem cells should therefore be susceptible one more time to androgen withdrawal. Thus, intermittent androgen blockade (IAD) would postpone the emergence of the androgen-resistant clone. In addition, preservation of quality-of-life of patients under IAD treatment will occur in terms of better tolerance of treatment; sometimes benefits in sexual function in off-treatment periods and reduction in the cost of treatment will be provided. However, IAD has not been reported to be related to prolonged hormone-sensitive status or an increase in OS (European Association of Urology Guidelines 2013).

We know that, currently, there is no standard front-line or second line therapy for these hormone refractory patients. For that reason, we must pay attention the patients’ quality-of-life when deciding on the method of treatment. At this point, the expectations of the patients are of great importance. In our opinion, treatment modalities that have reversible side effects should be chosen in order to evaluate the tolerance of the patients at the beginning of management. For that reason, medical treatment modalities should be chosen primarily in order to avoid the psychological side effects of surgery such as empty scrotum, hot flashing, etc.

Average survival of patients with metastatic PCa following relapse who do not undergo further treatment is 14 months (Matzkin et al. 1993). Unfortunately, despite all treatments, treatment failure occurs within 2 years of initial management. Treatment options for hormone refractory disease include intensive supportive care, radiotherapy, biphosphonates, second-line hormonal manipulations, cytotoxic chemotherapy and investigational agents. Chemotherapeutic agents have yielded improved response rates and palliative benefit, but not improved survival (Hussain et al. 1994; Murphy et al. 1983; Kantoff et al. 1999; Robinson et al. 1995). Therefore, new studies must focus on new agents in order to improve survival, time to progression and quality-of-life. We also believe that psychological support should be given at the time of progression to the hormone refractory stage for the sake of quality-of-life.

## Conclusion

As a conclusion; according to our results, there was no statistical difference between the hormonal treatments that were applied in metastatic prostate cancer in terms of the above mentioned parameters. Patients with androgen independent prostate cancer demonstrate progression of the disease despite chemical or surgical castration, and have poor prognosis.

## Methods

A database of 56 patients who had a metastatic PCa diagnosis and received hormonal therapy as initial management, which was applied until the emergence of hormone resistance, was retrospectively reviewed in order to understand the course of the disease. The patients were categorized into 3 groups according to the type of applied treatment modality. These groups comprised bilateral subcapsular orchiectomy (BSO) alone, bilateral subcapsular orchiectomy combined with anti-androgen therapy, and LH-RH combined with anti-androgen. One of these treatment modalities was applied to the patients as initial management. Serum PSA levels at the time of the first diagnosis, post-treatment nadir PSA levels, time to nadir PSA, time to hormonal resistance and PSA levels at hormonal resistance were assessed retrospectively. In addition to these, the localization and number of metastases and the term of survival from the beginning of the emergence of hormone resistance until death were investigated.

### Statistical analysis

One-way ANOVA test, a parametric statistical test, was used to compare the three groups with respect to the normally distributed variables in our data. A probability level of p < 0.05 was considered as significant.

## Authors’ original submitted files for images

Below are the links to the authors’ original submitted files for images. Authors’ original file for figure 1

## Abbreviations

Pca: Prostate cancer
PSA: Prostate specific antigen
ADT: Androgen deprivation therapy
MAB: Maximum androgen blockage
CAB: Complete androgen blockage
mCRPC: Metastatic castrate-resistant prostate cancer
BSO: Bilateral subcapsular orchiectomy
GS: Gleason score
ECOG: Eastern countries oncology group
SWOG: Southwest oncology group
AR: Androgen receptor
DES: Diethylstillbestrol
RCT: Randomized controlled trials
OS: Overall survival
IAD: Intermittent androgen blockade.
