# Supplementary material for: The course of metastatic prostate cancer under treatment
Source: Springerplus. 2014 Dec 10;3:725. doi: 10.1186/2193-1801-3-725 (PMC4320210; doi:10.1186/2193-1801-3-725)
Supplement: Supplementary file 1 — Authors’ original file for figure 1 [file 40064_2014_1491_MOESM1_ESM.doc]

| Parameters | | | | BSO n=12 | | BSO+AA n=18 | | LH-RH+AA n=26 | | WHOLE GROUP n=56 | |
| --- | --- | --- | --- | --- | --- | --- | --- | --- | --- | --- | --- |
|  |  |  |  | Median | Mean | Median | Mean | Median | Mean | Median | Mean |
| The PSA (ng/ml) values before teratment | | | | 91 | 171 | 143 | 383,7 | 115 | 626,5 | 110 | 423,8 |
| Gleason Score | | | | 7 | 7,33 | 7 | 7,5 | 7 | 7,52 | 7 | 7,4 |
| The nadir PSA (ng/ml) after treatment | | | | 1,3 | 31,01 | 3,6 | 37,47 | 2,4 | 7,87 | 2,71 | 21,68 |
| The time to the nadir PSA (months) | | | | 3 | 5,9 | 4,5 | 6,45 | 8,5 | 9,27 | 6 | 7,44 |
| The time to hormonal resistance (months) | | | | 18,7 | 32,77 | 17 | 26,48 | 21 | 27,3 | 20 | 30,28 |
| The PSA (ng/ml) at the hormonal resistance | | | | 103,5 | 124,2 | 54 | 163,7 | 38,5 | 890,6 | 54 | 489,9 |
| Survival (months) | | | | 25 | 45 | 47 | 45,25 | 32 | 34,35 | 34 | 42,67 |
|  |  |  |  |  |  |  |  |  |  |  |  |
| Table I: The summary of the results of each treatment group. All p values >0,05 | | | | | | | |  |  |  |  |
